# The Orbitofrontal Cortex Is Required for Learned Modulation of Innate Olfactory Behavior

**DOI:** 10.1523/ENEURO.0343-24.2024

**Published:** 2024-10-18

**Authors:** Kiana Miyamoto, Jeremy Stark, Mayuri Kathrotia, Amanda Luu, Joelle Victoriano, Chung Lung Chan, Donghyung Lee, Cory M. Root

**Affiliations:** Department of Neurobiology, School of Biological Sciences, University of California San Diego, San Diego, California 92093-0357

**Keywords:** aversion, innate, olfactory, orbitofrontal

## Abstract

Animals have evolved innate responses to cues including social, food, and predator odors. In the natural environment, animals are faced with choices that involve balancing risk and reward where innate significance may be at odds with internal need. The ability to update the value of a cue through learning is essential for navigating changing and uncertain environments. However, the mechanisms involved in this modulation are not well defined in mammals. We have established a new olfactory assay that challenges a thirsty mouse to choose an aversive odor over an attractive odor in foraging for water, thus overriding their innate behavioral response to odor. Innately, mice prefer the attractive odor port over the aversive odor port. However, decreasing the probability of water at the attractive port leads mice to prefer the aversive port, reflecting a learned override of the innate response to the odors. The orbitofrontal cortex (OFC) is a fourth-order olfactory brain area, involved in flexible value association, with behaviorally relevant outputs throughout the limbic system. We performed optogenetic and chemogenetic silencing experiments that demonstrate the OFC is necessary for this learned modulation of innate aversion to odor. Further, we characterized odor evoked c-fos expression in learned and control mice and found significant suppression of activity in the bed nucleus of the stria terminalis, lateral septum, and central and medial amygdala. These findings reveal that the OFC is necessary for the learned override of innate behavior and may signal to limbic structures to modulate innate response to odor.

## Significance Statement

This research establishes a new behavioral assay to train animals to reverse innate odor preference and establishes a role for the orbitofrontal cortex in updating innate valence upon experience. This provides an entry point for future work to further unravel the circuit mechanisms involved.

## Introduction

Animals have evolved sensory systems that afford innate and learned responses to stimuli in the environment, which allow an animal to respond to environmental cues that have been important for survival over its lifetime and over evolutionary time. This combination of innate and learned behaviors allows an animal to flexibly navigate changing landscapes that involve balancing risk and reward. Although innate behaviors such as feeding and mating are essential to species survival, they subject an animal to the risk of predation or other danger. Thus, innate drives must exist in a balance of risk and reward, which may be modulated by experience and internal need. We sought to elucidate the brain areas of the mouse involved in this executive override of innate odor-driven behaviors.

The sense of smell is essential for most animals and elicits innate behaviors including feeding, social behavior, and predator avoidance. Although these behaviors involve complex motor responses, they can be simplified into an axis of motivational valence (seeking or avoiding; Elliot, 2006, Tye, 2018), whereby an animal either approaches or avoids an odor. Olfactory perception begins with the recognition of odorants by a large repertoire of receptors in the sensory epithelium (Buck and Axel, 1991, Zhang and Firestein, 2002, Godfrey et al., 2004). Neurons expressing a given receptor are distributed within zones of the epithelium and project with precision to spatially invariant glomeruli in the olfactory bulb (Ressler et al., 1994, Vassar et al., 1994, Mombaerts et al., 1996). Functional imaging experiments reveal that odors activate spatial patterns of glomeruli, forming a stereotyped map in the first olfactory relay (Malnic et al., 1999, Rubin and Katz, 1999, Uchida et al., 2000, Wachowiak and Cohen, 2001), which can be readout to discriminate structurally similar odorants (Mathis et al., 2016). The activity of individual glomeruli is propagated by the second-order mitral and tufted cells to multiple third-order olfactory areas including the piriform cortex and cortical amygdala (Haberly and Price, 1977, Sosulski et al., 2011). Innate attraction and aversion to odor is mediated by the cortical amygdala (Root et al., 2014) by divergent projections to the medial amygdala and nucleus accumbens for avoidance and attraction to odor, respectively (Howe et al., 2024).

A few brain areas have been implicated in olfactory learning. The piriform cortex receives distributed and disordered input from the olfactory bulb (Sosulski et al., 2011) and has been implicated in pattern decorrelation and encoding odor identity (Stettler and Axel, 2009, Davison and Ehlers, 2011, Franks et al., 2011). It has also been thought to play a role in learning (Johnson et al., 2000), and other work has demonstrated the necessity and sufficiency for recall of learned associations (Choi et al., 2011, Meissner-Bernard et al., 2019). The ventral striatum has long been a focus for reward association, and the olfactory tubercle appears to play a role in encoding learned valence (Gadziola et al., 2015, Martiros et al., 2022, Lee et al., 2023). The OFC receives olfactory input from the piriform cortex (Clugnet and Price, 1987, C. F. Chen et al., 2014) and has been implicated in numerous cognitive functions including risk assessment and flexible behavior (Murray et al., 2007). Moreover, the OFC is known to represent value in both rodents and primates, and lesion experiments implicate the OFC in updating learned information (Schoenbaum et al., 2002, Gottfried et al., 2003, Murray et al., 2007, Balleine et al., 2011, Gottfried and Zelano, 2011). The representation of odor in OFC signals reward association and is required for reversal learning (Schoenbaum et al., 2002, Wang et al., 2019). Moreover, OFC has projections throughout the limbic system (McDonald et al., 1999, Gabbott et al., 2005) and is well positioned in the olfactory circuit to integrate olfactory information with value information to modulate innate odor valence through learning.

Here, we hypothesized that one function of OFC could be to modulate innate olfactory behaviors by reassigning odor valence akin to its role in reversal learning (Schoenbaum et al., 2002). We explored this possibility by creating a new olfactory assay that challenges a thirsty mouse to choose an aversive odor over an attractive odor in foraging for water. Learning led them to prefer the innately aversive odor over the attractive odor and inhibition of the OFC with either optogenetics or chemogenetics caused animals to revert back to their innate preference between these odors after learning. Moreover, we explored changes in odor evoked activity in limbic brain areas in response to the aversive odor. Learning led to a suppression of c-fos activity in the bed nucleus of the stria terminalis (BNST), lateral septum (LS), central amygdala (CeA), and medial amygdala (MeA). Thus, the OFC is required for learned modulation of innate olfactory behavior and may signal to limbic areas to suppress the aversive responses to modulate the behavior.

## Materials and Methods

### Experimental subjects

All procedures were approved by the University IACUC. Adult C57BL/6J male mice (Jackson Laboratory) aged 8–20 weeks were grouped housed until surgery and then singly housed on a reverse light cycle. For mice that did not require surgery, mice were singly housed at least 5 d prior to behavior. Optogenetic and chemogenetic mice were allowed 3 weeks of recovery from surgery. During behavioral training, mice were water restricted for 2 d prior to the start of training with only 1 h access to *ad libitum* water per day during training assay. Animals were weighed daily to maintain at least 80% of body weight.

### Stereotactic surgery

Animals were anesthetized with isoflurane (3% for induction, 1.5–2.0% afterward) and placed in a stereotaxic frame (Model 1900, Kopf Instruments). Animals were administered Ethiqa XR analgesic at the start of surgery. Blood oxygenation, heart rate, and breathing were monitored throughout surgery, and body temperature was regulated using a heating pad (PhysioSuite, Kent Scientific). A small craniotomy above the injection site was made using standard aseptic technique. The viral vectors (800 and 500 nl for eNpHR3.0 and hM4D, respectively) were injected into each hemisphere of OFC with needles pulled from capillary glass (3-000-203-G/X, Drummond Scientific) at a flow rate of 2 nl/s using a micropump (Nanoject III, Drummond Scientific). The viral vectors used were the following: AAV5-syn-eNpHR3.0-eYFP, AAV5-syn-eYFP (UNC vector core), AAV5-syn-hM4D(Gi)-mCherry, and AAV5-mCherry (Addgene). The stereotaxic coordinates used were +2.7 AP, −2.4 DV, and +/−1 ML, relative to the bregma according to the Franklin and Paxinos atlas. For optogenetic experiments, 200 μm diameter, 0.37 NA fiber cannulas (R-FOC-L200C-37NA, RWD Life Science) were implanted 0.1 mm above injection site and cemented to the skull using light curing glue (Tetric EvoFlow, Ivoclar Group) followed by black Ortho Jet-dental cement (Lang Dental) to cover the remaining skull.

### Behavior assays

The two-port assay was custom fabricated as a box (9″L × 6″W × 8.5″H) with a removable top with a 1 inch hole for optic fiber passage in the center. The box was assembled with clear acrylic sides and a white acrylic bottom. One long side had cutouts for the ports (1″W × 1.5″H), located with the center 2.5″ from each end of the box and 1.75″ from the bottom. The ports were stand-alone pieces screwed into a 24″ × 24″ optical breadboard (MB2424, Thorlabs). Thus, the boxes could be easily removed and cleaned between animals, without disconnecting the ports from water/odor lines. Ports were assembled from acrylic parts to create a port 2.5″H × 1.75″W × 1.5″deep. The port is slightly larger than the box opening to allow easy alignment with the box. The sides of the port had an odor-in connector attached with threaded holes and Teflon tubing connectors screwed in ∼0.5″ from the top and a similar vacuum port 0.5″ from the bottom on the opposite side. The water delivery spout was inserted from the back. Spout was made from a 22 ga gavage needle (18061-22, Fine Science Tools), with a wire soldered to it, for connection to capacitance sensor (1129_1, Phidgets), and inserted through a threaded tubing adapter with epoxy to hold it within the adaptor. The spout/adapter was threaded through a hole centered in the port centered 1.75″ from the bottom. Lastly, IR emitter and Sensor (SEN-18772 and SEN-19018, SparkFun) were attached to both sides of the port at ∼1.75″ from the bottom. The entire box and breadboard were enclosed in light proof Black Hardboard (TB4, Thorlabs).

Odor was delivered through three-way solenoid valves (LHDB1223418H, The Lee) that redirected clear air through a 100 ml bottle containing 50 μl of 1:10 odorant in mineral oil, TMT (1G-TMT-97, BioSRQ) or 2PE (77861, Sigma) on a small piece of kimwipe. Air flow was delivered to the port at a rate of 0.3 l/min. When the odor was off (mouse not in port), clean air was directed to the port. A vacuum line removed air at a rate of 1 l/min for each port, which was meant to create a negative pressure to contain the odor in the port. Flow rates were controlled by gas mass flow controllers (Allborg). Solenoid valves were controlled through custom software written in LabVIEW (National Instruments), as well as collection of data regarding licking and time in port. Briefly, the IR beam break detected entry into the port and created a TTL pulse delivered to a transistor array (511-ULN2004A, Mouser Electronics) to power the valves. The IR beam break was used to calculate time in the port. The water was delivered by gravity-fed two-way solenoid valves (LHDB1242115H, The Lee). The capacitance sensor attached to the lick spout triggered a TTL pulse from a data acquisition device (10183B, Phidgets) to open the valve for 50 ms, delivering a droplet of ∼5 μl.

For behavioral training, mice were water restricted for at least 1 d, trained on a schedule with 1 h per day of training where water was only available in the training paradigm. Mice were weighed daily and all mice maintained at least 80% their initial body weight. The training schedule began with 1 d of no odor and equal availability of water at both ports (Stage 1), followed by 1 d with the introduction of odor with equal water to measure their innate response to odor (Stage 2), and then odor with unequal water availability (Stage 3) for up to 6 d. On the last day (Stage 4), a 10 min probe trial was used where odor was delivered but not water. During the chemogenetic experiments, training followed a similar schedule except that we introduced 5 min probe trials on alternating days of training that provide a potentially better comparison of their learning to the final probe trial.

The program controlling the behavior recorded the cumulative amount of time spent each port, in 5 min bins saved to a csv file. In the first experiments (Extended Data Fig. 1-2), we used the cumulative time in the first 10 min in calculation of the preference index; whereas in the chemogenetic experiment, we used only the first 5 min probe trials. We did not observe clear differences between these two approaches. The preference index was calculated as $\mathrm{PI}=2*\left(\frac{\mathrm{TMT}}{\mathrm{TMT}+2\mathrm{PE}}-0.5\right)$. This index takes the ratio of time spent in the aversive port (TMT) over the total time in both ports (TMT + 2PE). This ratio is adjusted to center around zero by subtracted 0.5 and scaled to a range of 1 to −1 by multiplying by 2. Thus, if an animal spent all of its port time in the TMT port, the PI would equal 1, whereas if it spent it in the 2PE port, the PI would equal −1, and equal time in both ports would equal 0.

During the habituation assay, mice were water restricted and given 1 h per day of *ad libitum* access to water in their home cage. They were exposed to passive TMT for 5 consecutive days, whereby they were placed in our two-port assay, the vacuum line was turned off, and TMT was pumped into the box through the odor port for 5 min. After 10 min they were returned to their home cage. The four-field olfactometer was used as previously described (Root et al., 2014). Briefly, a mouse was placed in the chamber in dark conditions for a 10 min baseline plus 15 min odor period. Odor was delivered by passing air through a 100 ml bottle with 1 μl of pure TMT. Tracking of the animal and control of the odor was controlled by custom software written in LabVIEW (Root et al., 2014). The response to odor was measured between minutes 12–22 of the odor period, using a custom script written in Igor Pro (WaveMetrics) to parse location over time and calculate the PI.

Go-no-go behavior was designed using single ports as above, with boxes that were the same dimensions as the two-port, except that the cutout for the single port was centered on one short side. A custom program written in LabVIEW controlled the behavioral assay. The training strategy involved three stages as originally described (Bodyak and Slotnick, 1999): Stage 1, 1 d of 30 min in which the mice could lick for water and water was given at 1 Hz in a progressive schedule of required licking. Stage 2, mice could only get water if odor was presented, with a progressive delay between odor and water up to 1 s. Stage 3, animals learned to lick to isoamyl acetate (306967, Sigma) and withhold licks to pinene (147524, Sigma) both diluted 1:100 in mineral oil. Animals would typically perform at least 200 trials in a 30 min session. The fraction correct licks were calculated as number of licks following the CS+ / total number of licks.

### Optogenetic experiments

Mice were habituated to handling and custom, homemade fiber-optic patch cords were attached to fiber cannulae and then connected to a 561 nM SLOC laser (Shanghai Laser & Optics Century) through a fiber-optic rotary joint (FRJ_1×2i_FC-2FC, Doric Lenses). The laser was turned on for 10 min at the start of the experiment, measuring 7–10 mW at the tip of each fiber patch cord. The radius of illumination is calculated with trigonometry using the half angle of divergence for a multimode optical fiber, $\theta =\sin^{-1}\;\left(\frac{\mathrm{NA}}{n}\right)$, where NA is the numerical aperture of the fiber (0.37) and *n* is the index of refraction of gray matter (1.36; Vo-Dinh, 2003). Power attenuation was calculated as described (Aravanis et al., 2007): $\frac{I_{z}}{I_{z0}}=\frac{\rho^{2}}{(S_{z}+1){\left(z+\rho \right)}^{2}}\;$, where $\rho =r\sqrt{{\left(\frac{n}{\mathrm{NA}}\right)}^{2\;}-1}$ and *S_z_* is the scatter coefficient (11.2) per unit thickness and *z* is the thickness of the sample. From this we estimate that the conical radius of illumination is 05.0.6 mm from the fiber time. The OFC is ∼0.8 mm long in the AP axis and ∼1.7 mm in ML axis at the center. Thus, with our fibers implanted at the midline along the AP axis and midline between ventral and lateral OFC, our optical inhibition should be largely restricted to the ventral and lateral portions of the OFC.

### Chemogenetic experiments

Mice were administered either saline (S5885, Teknova) injection or CNO dissolved in saline 3 mg/kg (HB6149, Hello Bio) by intraperitoneal injection 1 h prior to the start of behavior.

### Histology

Mice were administered ketamine (100 mg/kg) and xylazine (10 mg/kg) and killed by transcardial perfusion with 10 ml of PBS followed by 10 ml of 4% paraformaldehyde in PBS. Brains were extracted and left in a 4% PFA solution in PBS overnight. Then, 50 μm coronal sections were cut on a vibratome (VT1000, Leica). A subset of tissue was labeled using the following simplified staining protocol. First, brain sections were incubated for 24 h at 4°C in the primary antibody diluted 1:1,000 in PBST (0.5% Triton X-100 in PBS). Brain sections were then washed three times for 10 min in PBST before and after incubating for 2 h at room temperature in the secondary antibody diluted in PBST. The antibodies used in this study are the following: goat anti-GFP (ab6673, Abcam), alexa-488 donkey anti-goat (A11055, Life Technologies), rabbit anti DSRed (632496, Clontech Labs 3P), rabbit anti c-fos (2250S, Cell Signaling Technology), alexa-568 donkey anti-rabbit (Life Technologies, A11057). Slices were mounted using Fluoromount-G with a DAPI counterstain (0100-20, SouthernBiotech) and imaged on an Olympus BX61 VS120 Virtual Slide Scanner and 10× objective (Olympus).

## Results

### Behavioral paradigm for the modulation of innate olfactory behavior

**Figure 1.**
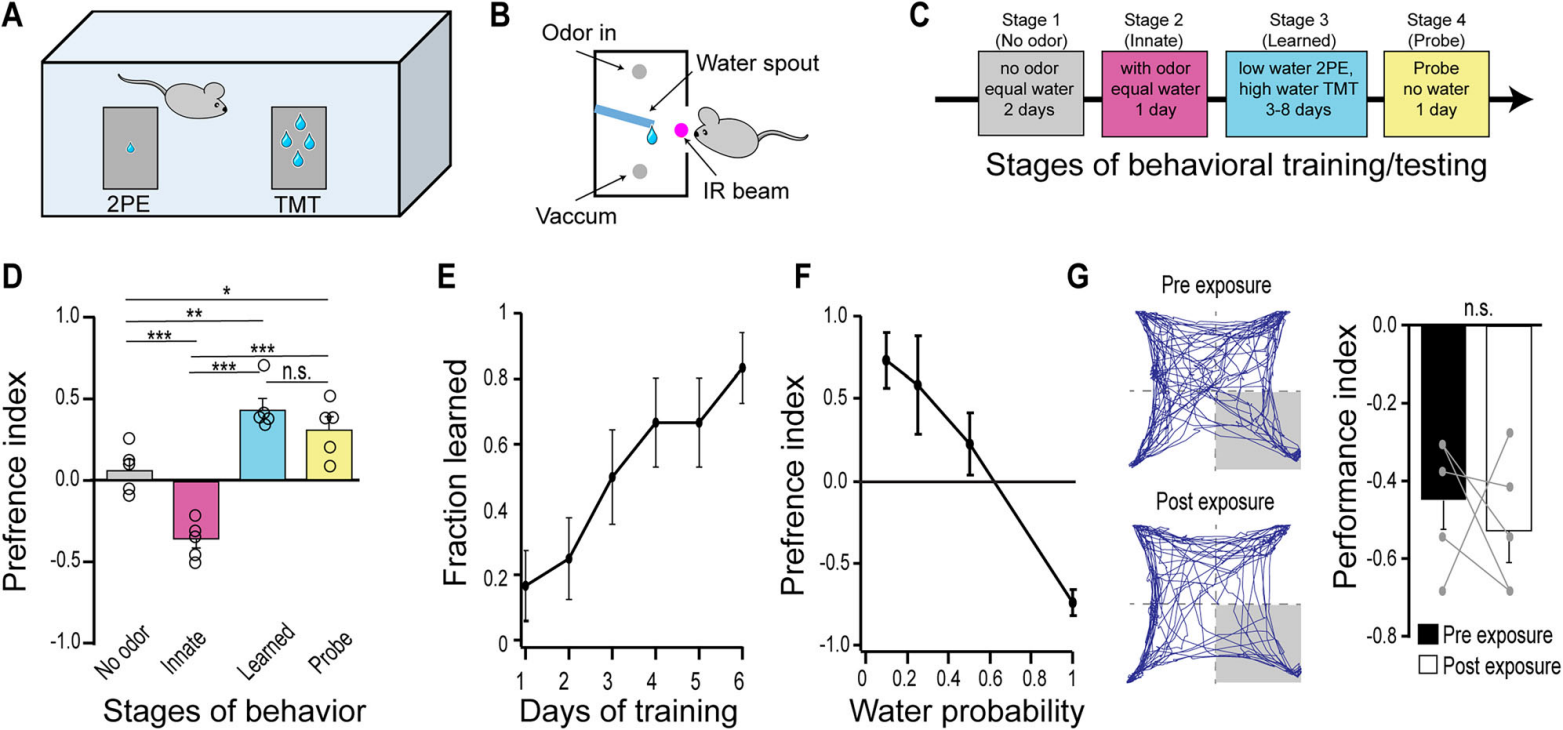
A behavioral paradigm for modulating innate response to odor with experience. ***A***, Two-port behavioral assay to pair odor with variable amount of reward during association. ***B***, Side view of the odor port design. Odor comes in at the top and a vacuum line pull odor out at the bottom. An IR beam and sensor are positioned at the entrance of the port to turn on odor and a water spout connected to a capacitance sensory delivers water droplets at set frequency or probability upon licking. ***C***, Training schedule: Stage 1, train animals to drink from port; Stage 2, measure innate response to odor; Stage 3, train mice with higher probability of water at the port with aversive odor; Stage 4, probe trials to measure response to odor in the absence of water. ***D***, Port preference across stages of training quantified with preference index $=2*\left(\frac{\mathrm{TMT}}{\mathrm{TMT}+2PE}\right)-0.5)$. ***E***, Fraction of mice learned to criterion across days. ***F***, Preference index as a function of the probability of water delivery at the 2PE port. ***G***, Test for habituation to TMT. A separate cohort of mice were tested in a four-quadrant arena (Root et al., 2014) before and after 5 d of passive TMT exposure. Left, Trajectory of a single mouse in response to minutes of TMT delivered to the right quadrant before (top) and after (bottom) 5 d of passive exposure to TMT. The avoidance measured with a performance index, PI=(P−25)/0.25, where *p* is the percentage of time in the odor quadrant. PI of 0 is neutral and negative values are aversive. Paired *t* test; n.s., not significant; *p < 0.05, **p < 0.01, ***p < 0.001. *n* = 5–10 each group.

We began by creating a behavioral paradigm that resembles a risk–reward tradeoff in a foraging task. In this assay, a water-restricted mouse is introduced into a chamber with two water ports that have either the innately aversive odor, 2,3,5-trimethyl-3-thiazoline (TMT), or the attractive, 2-phenylethanol (2PE; Fig. 1*A*). Each port was equipped with an IR beam detector to detect port entry, a water spout with a contact sensor to deliver water droplets at 0.1–1 Hz. Odor delivery was controlled by solenoid valves that open upon IR beam break, while a vacuum line removed odor from the port (Fig. 1*B*). Mice were water restricted and trained on a schedule with 1 h per day of training where the availability of water could vary between the ports (Fig. 1*C*). The training schedule began with no odor and equal availability of water at both ports (Stage 1), followed by the introduction of odor with equal water to measure their innate response to odor (Stage 2). We calculated a preference index that is the ratio of time in the TMT port over the total time in both port that is scaled to center between −1 and 1 $\left[\mathrm{PI}=2*\left(\frac{\mathrm{TMT}}{\mathrm{TMT}+2\mathrm{PE}}-0.5\right)\right]$, whereby negative values indicate a preference for 2PE and positive values indicate preference for TMT.

In the absence of odor, mice explore both ports equally, but introduction of odor causes them to spend more time in the 2PE port, reflecting their innate response (Fig. 1*D*). Next, we challenged them to spend more time in aversive TMT port by decreasing the availability of water at the 2PE port (1 Hz drops at TMT vs 0.1 Hz at 2PE; Stage 3). During this training the odor ports were reversed daily to prevent association with a location. This training led them to learn the association between odor and water that persisted in probe trials (Stage 4) where they explored the ports with no water available. We considered them to have learned the association by spending 1.5× more time in the TMT port than the 2PE port (PI >0.2). With this criterion, we assessed the odor preference as a function of training days and found that after 3 d approximately half of the mice learned the association and by 6 d of consecutive training days 83% of animals learned (Fig. 1*E*). Next, we considered how the learning might change as a function of water availability. We tested this by modifying the water delivery to be at a potential rate 1 Hz for both ports but with decreasing probability at the 2PE port (probability of 0.1 to 1). We observed that probabilities below 0.5 at the 2PE port led mice to prefer the TMT port (Fig. 1*F*), whereas equal probability led to preference for 2PE.

Repeated exposure to odor changes the response of sensory neurons (Tsukahara et al., 2021), suggesting the possibility that habituation could play a role in our assay. Although it is not known whether mice habituate to TMT with repeated exposure, we wondered if the apparent attraction to TMT could be in part due to habituation of response to TMT. We investigated this by passively exposing a naive cohort of mice to TMT daily for comparable amounts of time (twice the median amount of time mice spent in the TMT port during learning, 5 min), in the training boxes without water across 5 d. Mice were water restricted to state match the conditions but did not receive water associated with TMT. We measured and compared their avoidance with TMT in a four-field olfactory assay (Root et al., 2014) and found that 5 d of passive TMT exposure did not affect their avoidance (Fig. 1*G*), indicating that habituation to the odor does not contribute to the behavior seen in our learning assay and suggests more generally that habituation to innately relevant odors might not occur.

### The OFC is necessary for modulation of innate response

**Figure 2.**
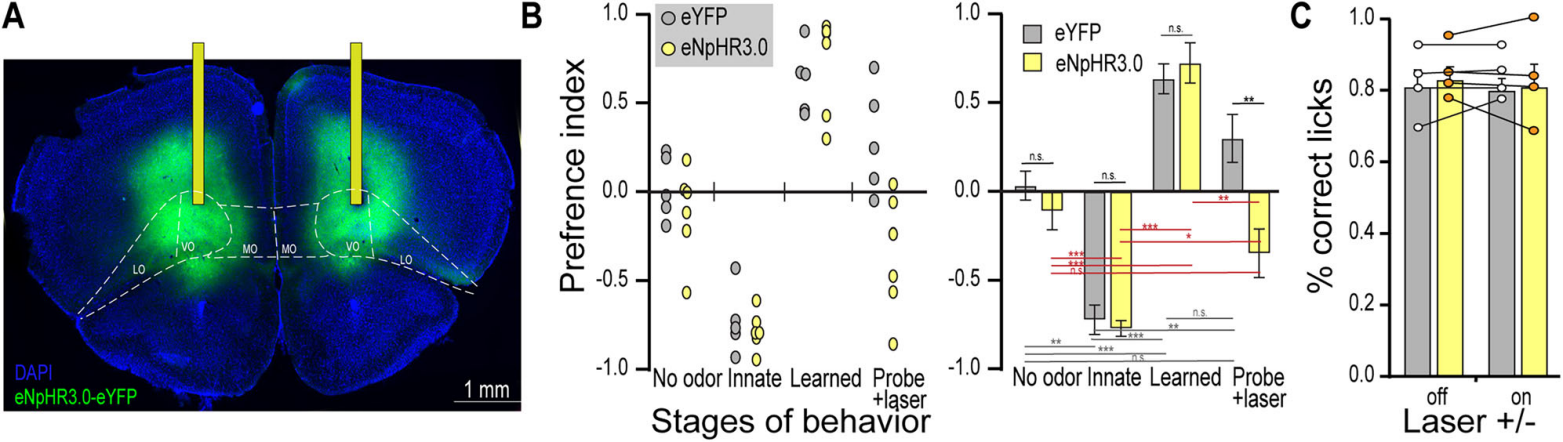
Optogenetic silencing of the OFC prevents expression of learned override of innate behavior. ***A***, Representative histology from bilateral expression of eNpHR3.0-eYFP in the OFC with optic fibers implanted above the region. The regions of OFC are outlined as medial (MO), ventral (VO), and lateral (LO) according to Franklin and Paxinos. See Extended Data Figure 2-1 for more details. ***B***, Mice were tested in the two-port assay to measure the preference for TMT versus 2PE across stages of behavior. The probe trial included 10 min of optical stimulation. The left plot shows performance of individual animals, and the right shows the mean with statistical analysis. Statistics: top bars, unpaired *t* test between groups. Bottom, paired *t* test between stages gray bars for control and red bars for halo animals. n.s., not significant, *p < 0.05, **p < 0.01, ***p < 0.001, *n* = 5–6 each group. ***C***, Mice were tested in a go-no-go simple association task and the performance as % correct licks is plotted with and without optical stimulation. Paired and unpaired *t* tests found no significant differences, across conditions or group, *n* = 4 each group.

The OFC has been shown to be involved in flexible value assignment for odor and we hypothesized that the OFC could play a role in this learning paradigm. Therefore, we used optogenetics to silence the OFC with bilateral halorhodopsin expression. Mice were bilaterally injected with AAV-syn-eNPHR3.0, or AAV-syn-eYFP as a control, and optic fibers were implanted above the OFC. Our injections mainly targeted the ventral OFC, with some expression in the lateral portion (Fig. 2*A*). The labeling spread ∼400–500 μm from the center in both directions along the anterior–posterior axis, covering a large extent of the ventral and medial portion of lateral OFC. Placement of the fibers was documented (Extended Data Fig. 2-1), and the conical radius of illumination was estimated to be ∼0.5–0.6 mm. Given that the OFC is ∼0.8 mm long in the AP axis and ∼1.7 mm in ML axis at the center, our fibers implanted at the midline between ventral and lateral OFC, our optical inhibition should be largely restricted to the ventral and lateral portions of the OFC. Mice were allowed 3 weeks to recover from surgery and for viral expression. Next, mice were subjected to the above training schedule (Fig. 1*B*) and trained until they had reached criterion for two consecutive days and then tested in 10 min probe trials without water, and 561 nm light (7–10 mW) was delivered. We observed that this suppression caused animals to reverse back toward the innate preference for the 2PE, whereas control animals exhibited the learned preference for the TMT port (Fig. 2*B*). The port preference is somewhat reduced in these trials compared with the learning phase because water was removed to examine only the odor response, consistent with what was observed in wild-type controls (Fig. 1). Thus, these optogenetic experiments indicate that the OFC is required for the display of a learned modulation of innate response to odor.

10.1523/ENEURO.0343-24.2024.f2-1 Figure 2-1 **Targeting for optogenetic experiments.** Schematic of the OFC at approximately 2.5 mm anterior to bregma according to Franklin and Paxinos, which is the midpoint along the anterior-posterior axis. Colored circles indicate the placement of bilateral fibers in each of six animals, whereby one color represents each animal. Fibers were annotated at approximately 2.4-2.6 mm from Bregma. The subregions of OFC are outlined as medial (MO), ventral (VO) and lateral (LO). The fibers were targeted to the boundry of VO and LO and it is estimated that the optical illumination spread 0.4 mm radius from the fiber tip. Download Figure 2-1, TIF file.

We next wanted to investigate the possibility that silencing the OFC could nonselectively interfere with their ability to smell or form simple odor associations. Therefore, we tested the same animals in an olfactory go-no-go discrimination assay, whereby mice learned to lick to isoamyl acetate and withhold licks to pinene, two innately neutral odors. After animals learned this task above 70% correct trials, we optogenetically silenced the OFC as above and observed that this manipulation did not significantly affect their performance in the odor discrimination task (Fig. 2*C*), consistent with previous findings (Schoenbaum et al., 2002). Thus, silencing the OFC does not broadly impair the sense of smell or learning but does implicate the OFC circuitry in modulation of innate responses.

**Figure 3.**
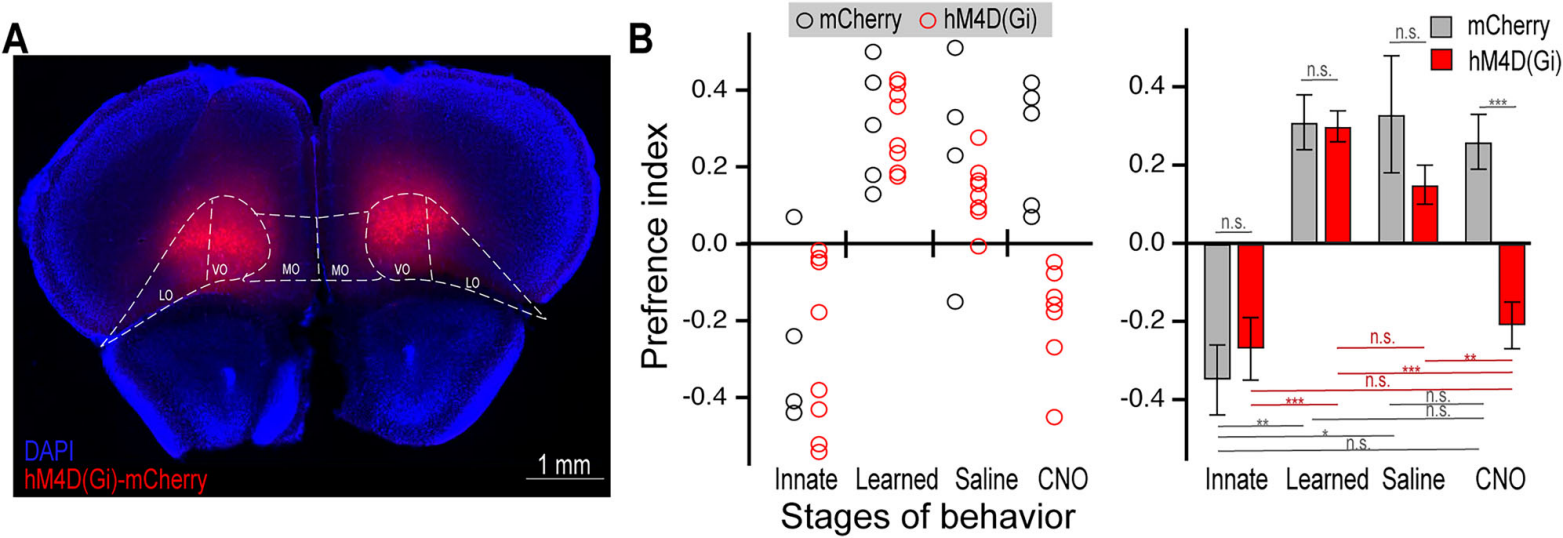
Chemogenetic silencing of the OFC prevents expression of learned behavior. ***A***, Representative histology from bilateral expression of hM4D(Gi)-mCherry in the OFC. The regions of OFC are outlined as medial (MO), ventral (VO), and lateral (LO) according to Franklin and Paxinos. See Extended Data Figure 3-1 for more details. ***B***, Mice were tested in the two-port assay for preference between TMT and 2PE across stages of behavior. In this experiment, 5 min probe trials were implemented in alternating days training and each stage shown is a probe trial. The left plot shows performance of individual animals, right and the shows the mean with statistical analysis. Statistics: top bars, unpaired *t* test between groups. Bottom, paired *t* test between stages, gray bars for control and red bars for hM4D(Gi) animals. n.s., not significant, *p < 0.05, **p < 0.01, ***p < 0.001, *n* = 5–8 each group.

Although the optogenetic silencing of OFC had a significant effect on modulation of innate response, we sought to replicate this finding with a different method of neuronal silencing. In particular, we were concerned about the long light stimulation that has the potential to cause heat damage. Therefore, we next employed the chemogenetic receptor, hM4D(Gi) (Armbruster et al., 2007), to inhibit OFC upon administration of the ligand, clozapine *N*-oxide (CNO). Mice were bilaterally injected with AAV-syn-hM4D-mCherry or AAV-syn-mCherry as a control (Fig. 3*A*). Viral injections were of a smaller volume than above to more carefully limit expression to OFC. The spread of infection was 200–400 μm from the center of OFC in both directions along the anterior–posterior axis and was largely limited to the ventral and lateral portions of OFC (Extended Data Fig. 3-1). We modified the training schedule to introduce 5 min probe trials on alternating days of training. In these experiments, we only considered their preference during these probe trials. Mice were trained to criterion and then given probe trials with either saline or CNO injection. These animals exhibited normal innate responses when there was equal water at both ports and then learned to prefer the TMT port, which was not significantly different between groups. Administration of saline did not significantly change their preference for TMT. In contrast, animals expressing hM4D(Gi) reversed toward there innate preference for 2PE upon CNO administration, whereas controls were unaffected (Fig. 3*B*). Thus, chemogenetic silencing of the OFC prevented the learned modulation of innate response.[Fig eN-NWR-0343-24F4]

10.1523/ENEURO.0343-24.2024.f3-1 Figure 3-1 **Targeting for chemogenetic experiments.** Representative histology from bilateral expression of hM4D(Gi)-mCherry in the OFC Mice along the most of the extent of OFC from the anterior (2.8 mm anterior to bregma) to the posterior (2.0 mm anterior to bregma). The top and bottom represent the extremes for viral spread, whereby the top set of images are from on mouse with the largest spread of virus, and the bottom are from a mouse with the smallest spread. Note, the red signal around the perimeter of some bottom images is background fluorescence, or imaging artifact, not mCherry expression. Download Figure 3-1, TIF file.

**Figure 4. eN-NWR-0343-24F4:**
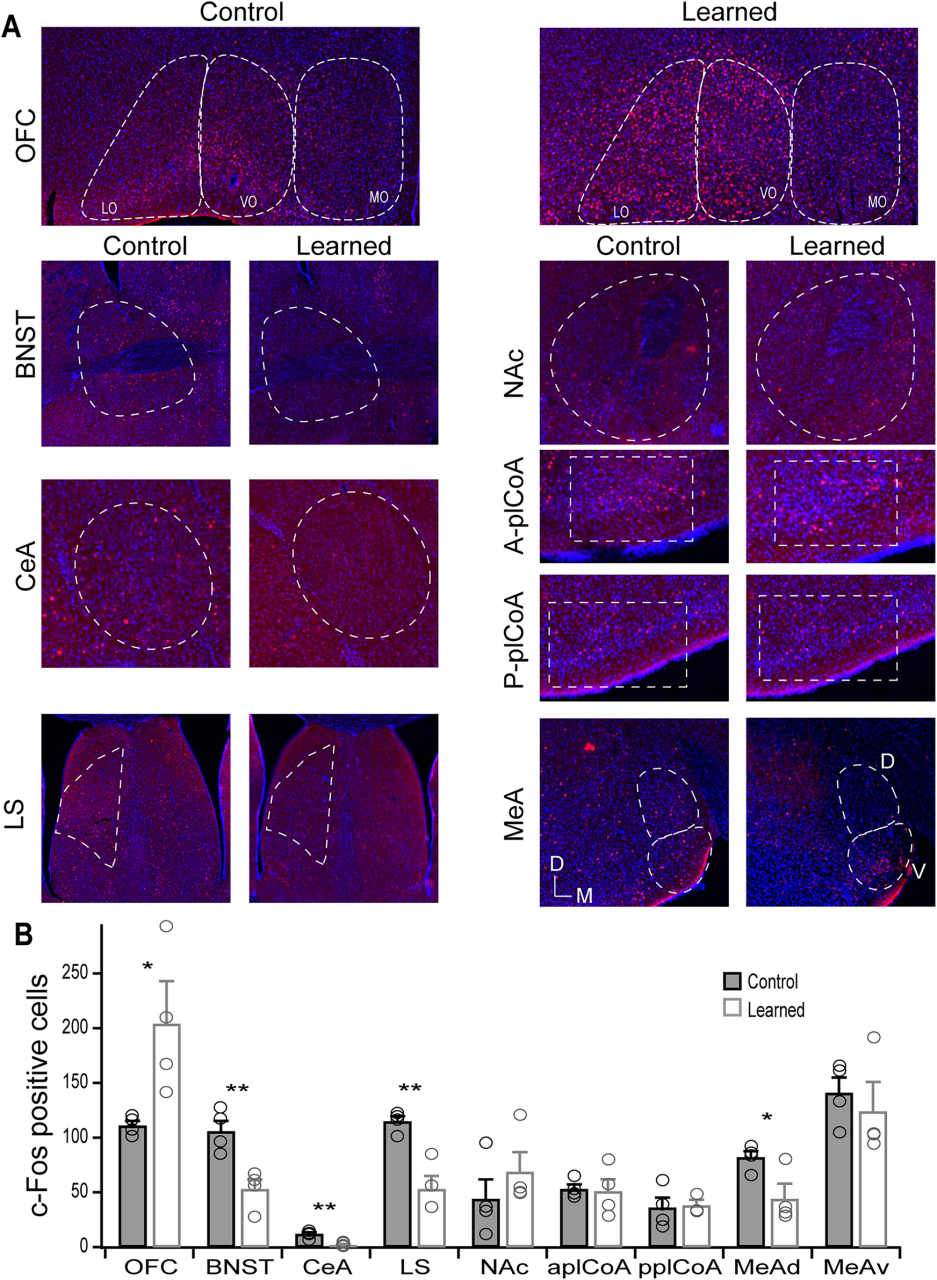
Mapped changes in neural activity after learned override of innate behavior. Two groups of mice were exposed to TMT and assayed for c-fos expression. One group went through Stages 1–3 of training, and a control group was water deprived and handled similarly with no exposure to TMT. ***A***, Representative images of c-fos activity in the OFC and seven limbic brain regions for control and learned mice. ***B***, Quantification of c-fos activity as the total number counted in each area per section. Counts from individual animals are overlaid as circles. Unpaired *t* test, *p < 0.05, **p < 0.01, *n* = 4 each group.

Lastly, we wondered what downstream brain areas might be modulated during learning. The OFC is known to have distributed projections throughout much of the brain, including amygdala and extended amygdala regions (Groenewegen et al., 1990). Thus, we focused on areas known to be part of the innate olfactory pathway as well as areas associated fear and anxiety. To assess changes in neural activity, we employed the immediate early gene, c-fos, as a marker for odor-activated neurons. We trained one cohort of mice using the schedule in Figure 1*B*, and a control group was water restricted and placed into parallel training boxes without any odor, just water (Stage 1) for the training duration of the learning group. After the learning group had reached criterion for at least 2 d, both groups were exposed to TMT in the training boxes without water for 5 min, akin to probe trials. It is noteworthy that c-fos labeling in the learned group may also capture response to the probe trial itself, in which TMT is being devalued by the absence of water, but we note that during the probe trial mice display a modulation of their innate response. Mice were killed 1–1.5 h later and processed for c-fos immunohistology. We surveyed a select group of brain areas that could be involved in innate response to odor (Fig. 4*A*). The cortical amygdala (plCoA) mediates innate responses (Root et al., 2014) and has projections of interest to the BNST, LS, MeA, and NAc. Of particular interest, projections from the NAc and MeA have recently been shown to mediate approach and avoidance responses to odor (Howe et al., 2024). Further, the plCoA was also found to have a topographic organization for valence, whereby anterior and posterior regions contribute to approach or avoidance responses. Therefore, we quantified the number of c-fos expressing neurons in these brain areas in learned and control mice. Strikingly, we found that learning led to a suppression of activity in a dorsal anterior region of the BNST surrounding the anterior commissure, CeA, LS, and dorsal MeA (Fig. 4*B*). No significant changes were seen in the anterior or posterior plCoA, ventral MeA, or NAc, though the NAc showed a trend toward increased c-fos activity. It is possible that the small sample size lacks the statistical power to detect smaller changes. These results indicate that areas downstream of the cortical amygdala implicated in fear, anxiety, and avoidance are suppressed upon learned modulation of innate response to odor. These observations do not rule out the possibility that relevant changes occur in other brain areas but demonstrate changes in areas downstream of the cortical amygdala innate pathway. It remains to be determined if the OFC has direct projections to these structures and is responsible for their modulation in activity.

## Discussion

Our findings demonstrate that mice can learn to override their innate responses to odor in a state-dependent water foraging task. The OFC plays a role in this, as inhibition of the OFC reverts their behavior toward the innate odor preference. Lastly, we observed changes in odor evoked activity following learning, whereby increased activity in the ventral and lateral portions of OFC are accompanied by suppressed activity in parts of the innate olfactory pathway that include the MeA and BNST as well as other areas associated with fear, anxiety, and avoidance.

The finding that learning leads to less TMT-evoked activity in the MeAD indicates that there is a suppression of the innate aversive pathway. Recent work demonstrated that the plCoA mediates innate aversive responses via a projection to the MeA (Howe et al., 2024). We did not find any changes in c-fos activity in the plCoA, which suggests that the first point of modulation in the innate aversive pathway is the MeA. The BNST and LS are targets of the plCoA, and their contribution to innate responses remains unclear, though silencing the BNST suppresses TMT-induced freezing behavior in rats (Fendt et al., 2003) and the LS has been implicated in sustained anxiety (Anthony et al., 2014). The CeA is well characterized to mediate aversive learning as the output of the basal lateral amygdala but is not implicated in TMT response (Fendt et al., 2003). Although the innate olfactory pathway is not fully resolved, our data indicates a suppression at two sights of this pathway, the MeA and BNST.

We reason that this override of innate behavior might involve suppression of an innate pathway and recruitment of an appetitive pathway. Is there a brain area that signals positive valence that has increased activity upon learning? We hypothesized that the NAc could be a sight for recruiting a positive valence as this is part of the innate approach pathway (Howe et al., 2024). However, we found an insignificant trend of increased activity, perhaps limited by the small sample size. It is possible that a more thorough characterization of activity in the NAc or another brain area might reveal significantly increased activity upon learning. It is important to note that we did not assess changes in activity evoked by 2PE. Given that the innate attraction to 2PE is also suppressed in the context of this behavior, it would be expected that some brain areas should also be modulated in to suppress attraction.

The frontal cortex has previously been found to play a role in updating value with experience. The mPFC appears to inhibit fear responses in the amygdala during fear extinction (Milad and Quirk, 2002). The OFC also plays a role in conditioned taste aversion, in which pairing of sucrose with lithium chloride causes aversion to the innately attractive sucrose (Ramirez-Lugo et al., 2016). This conditioned taste aversion leads to the recruitment of an aversive pathway in the parabrachial nucleus (J. Y. Chen et al., 2018) without any known change in the sweet pathway (Yamamoto et al., 1994). Our modulation of innate behavior is conceptually similar to both of these examples, whereby frontal cortex might modulate downstream areas, either directly or indirectly, to alter the valence of a sensory cue, but different in that the valence is reversed rather than suppressed. We propose a model whereby OFC projections to limbic structures such as the MeA and BNST suppress innate aversion and an unidentified projection may link the aversive odor to an appetitive output. However, further work is necessary to validate this hypothetical model.

## Synthesis

Reviewing Editor: Timothy Jarome, Virginia Polytechnic Institute and State University Decisions are customarily a result of the Reviewing Editor and the peer reviewers coming together and discussing their recommendations until a consensus is reached. When revisions are invited, a fact-based synthesis statement explaining their decision and outlining what is needed to prepare a revision will be listed below. The following reviewer(s) agreed to reveal their identity: Ryan Parsons.

Both reviewers agreed that the manuscript is novel, the study well controlled and data well presented. However, several concerns were raised that need addressed before the manuscript can be considered for publication.

1. Some changes to Figure 1 might be helpful. The timeline refers to numbered stages of behavioral testing, but the graphs (Fig1D-F) depicting the data from those stages do not refer to them by number.

2. The images depicting viral injections, especially in the optogenetic experiment, show a large amount of expression outside of the OFC making it difficult to know if the effects are the result of inhibition of OFC or adjacent areas. First, some discussion of this limitation would be useful and whether there are any data that would argue for an effect of OFC versus PL or other regions. Second, a reconstruction of the spread across the anterior-posterior extent of the expression for all animals (or perhaps showing largest and smallest amount of expression) is necessary.

3. From the optogenetic and chemogenetic experiments, data clearly indicates that inhibition of OFC neurons is necessary to reduce the preference, however whether their activation is sufficient, is not clear. Addition of these data will enrich the manuscript.

4. In the cFos experiments, trained mice are killed after the probe test and compared to untrained animals that also receive TMT during the probe. This controls for learning and exposure to TMT, but it is unclear if changing the outcome of responding (i.e. withholding the water) during the probe test might drive neural activity rather than, or in addition to, expression of the learned behavior.

5. The cFos experiment only looks at neural activity in select downstream regions of the OFC. Focusing on these regions is reasonable, but it would be useful to also analyze cFos expression in the OFC itself, especially since the opto/chemo studies target the OFC. It is also recommended to include the cFos data for 2PE control group.

6. Although cFos data indicate how differentially several brain regions responding to the TMT and learning, more discussion needed to downstream inputs from OFC and its correlation to the valence updating.5. The sex of the mice was not stated.

7. The sample size (e.g. N=4) is too low for the results to be reliable for some of the experiments.

8. Methods are missing details of the task and there's no description of the data analysis except in the figure captions. Some details of the task are offered in the results section, but it's a bit cumbersome to have it organized this way.

9. Please add the subheading in the result section for clarity.

10. It is advised to indicate the individual data points in graphs to appreciate data distribution.

## Author Response

We thank the editor and reviewers for their comments and sugges5ons. Our responses to individual points are below.

Synthesis Statement for Author (Required):

Both reviewers agreed that the manuscript is novel, the study well controlled and data well presented. However, several concerns were raised that need addressed before the manuscript can be considered for publica5on.

1. Some changes to Figure 1 might be helpful. The 5meline refers to numbered stages of behavioral tes5ng, but the graphs (Fig1D-F) depic5ng the data from those stages do not refer to them by number.

Thank you for the sugges5ons. We have changed the axis label for Fig1C to beMer match 1D. and clarified in the legend that 1E&F are during stage 3. Fig1E shows the frac5on that learned as a func5on of number of days in stage 3. Fig1F shows the preference index as a func5on of water probability on day 6 of stage 3.

2. The images depic5ng viral injec5ons, especially in the optogene5c experiment, show a large amount of expression outside of the OFC making it difficult to know if the effects are the result of inhibi5on of OFC or adjacent areas. First, some discussion of this limita5on would be useful and whether there are any data that would argue for an effect of OFC versus PL or other regions. Second, a reconstruc5on of the spread across the anterior-posterior extent of the expression for all animals (or perhaps showing largest and smallest amount of expression) is necessary.

This is an important issue. Indeed, the spread of viral labeling in the optogene5c experiment is broad (about 0.4-0.5mm radius from the injec5on target in the center of OFC). This was inten5onal, reasoning that the manipula5on is ul5mately restricted by the cone of light emiMed from the op5c fiber. We have now documented the placement of the fibers in Supplementary Figure 1a, and es5mated the conical radius of illumina5on based on published calibra5ons, to be about 0.5-0.6 mm (lines 181-190). The OFC is about 0.8 mm long in the AP axis and about 1.7 mm in ML axis from the center. Thus, with op5c fibers implanted at the mid line along the AP axis and midline between ventral and lateral OFC, our op5cal inhibi5on should be largely restricted to the ventral and lateral por5ons of the OFC. We do not observe much if any labeling of PL, and the photos5mula5on is not expected to reach it with sufficient intensity. Thus, it is expected that our manipula5on should be largely restricted to this por5on of the OFC.

As for the chemogene5c experiment, the infec5on volume was inten5onally less to limit the spread to only OFC (since light couldn't be used to further control inhibi5on). We have reviewed all of the histology and now present the two extremes for least and most amount of spread along the OFC axis. We find that the spread is indeed largely restricted to the ventral and lateral por5on of the OFC, with some expression in medial OFC. Thus, this manipula5on is unambiguously restricted to parts of the OFC, though not spanning its en5rety.

In regard to both of these experiments, we have also provided beMer discussion of the targe5ng and acknowledged poten5al limita5ons (lines 260-266, 290-294).

3. From the optogene5c and chemogene5c experiments, data clearly indicates that inhibi5on of OFC neurons is necessary to reduce the preference, however whether their ac5va5on is sufficient, is not clear. Addi5on of these data will enrich the manuscript.

The reviewer raises an interes5ng ques5on. We agree that an ac5va5on of OFC would enrich the story, by tes5ng if they are sufficient to modulate learning and/or innate behavior. However, it is unclear which neurons of OFC would be appropriate to ac5vate. Indeed, we had tried such an experiment using a c-fos trap approach but it failed. The idea was to capture neurons ac5vated by TMT acer learning and ask if ac5va5ng them could either suppress TMT avoidance in another assay or replace TMT odor in probe trial. Although the results were nega5ve, there were unresolved technical concerns that make us unsure of the findings, so we do not wish to include it. Addi5on of such an experiment would require considerable effort and is beyond the scope of this publica5on.

4. In the cFos experiments, trained mice are killed acer the probe test and compared to untrained animals that also receive TMT during the probe. This controls for learning and exposure to TMT, but it is unclear if changing the outcome of responding (i.e. withholding the water) during the probe test might drive neural ac5vity rather than, or in addi5on to, expression of the learned behavior.

If we understand correctly, the reviewer is sugges5ng that c-fos labeling in the learned animals may be par5ally capturing the effect of withholding water during the probe trial and not just the learning, in other words a devalua5on of TMT. This is an interes5ng idea that we hadn't considered. We note that during the probe trial mice s5ll prefer the TMT port over the 2PE port, so it is expected that the ac5vity is at least partly relevant to this modula5on of the innate response. Nonetheless we cannot rule out the poten5al that some neuronal ac5vity results from the devaluing of cue. We have added an acknowledgment of this more nuanced point (lines 312-314).

5. The cFos experiment only looks at neural ac5vity in select downstream regions of the OFC.

Focusing on these regions is reasonable, but it would be useful to also analyze cFos expression in the OFC itself, especially since the opto/chemo studies target the OFC. It is also recommended to include the cFos data for 2PE control group.

We have included analysis of the OFC as suggested for TMT exposure finding a significant increase in c-fos acer learning (Figure 4). Unfortunately, we do not have data for 2PE, and repea5ng the experiment is not feasible at the moment. Although this would be interes5ng to include, we think this is a new ques5on beyond our focus on the suppression of the innate avoidance. We have added a note that response to 2PE is also modulated in that aMrac5on is suppressed and it is possible that response to 2PE is also modulated (lines 358-361).

6. Although cFos data indicate how differen5ally several brain regions responding to the TMT and learning, more discussion needed to downstream inputs from OFC and its correla5on to the valence upda5ng.

Our targets for c-fos analysis were based on our understanding of the aversive pathways for TMT, more than the projec5ons of OFC. Thus, having iden5fied sites of modula5on in the innate pathway, we speculate that OFC may intersect these sites, either directly or indirectly. We have added a statement that "OFC is known to have distributed projec5ons throughout much of the brain including amygdala and extended amygdala" and clarified that we focused on areas known to be involved in TMT pathway or generalized fear and anxiety. (lines 304-307). We acknowledge that it remains to be determined if OFC has direct projec5ons to these areas (line 331-332) and that the modula5on of these brain areas may be direct or indirect (line 370).

5. The sex of the mice was not stated.

We have included in the methods that the mice were all male. (line 80) 7. The sample size (e.g. N=4) is too low for the results to be reliable for some of the experiments.

Two experiments have n=4. The first is optogene5c silencing of OFC in Go-No-Go simple associa5on task. This result simply confirms what was observed in classic lesion experiments, and there is liMle variance. Thus, there is liMle reason to think a larger sample size would change the outcome. The second is the c-fos experiment. Although the sample size is small in this instance, the variance is low, the data are normally distributed, and significant findings were detected. If one were to repeat the experiments assuming a similar distribu5on, it is unlikely that different conclusions could be drawn regarding the significant findings. However, it is possible that we do not have the sta5s5cal power to detect smaller effects in other areas. To address this, we have acknowledged the small sample size poten5ally obscures more subtle effects. (Lines 326-327, 356-357) 8. Methods are missing details of the task and there's no descrip5on of the data analysis except in the figure cap5ons. Some details of the task are offered in the results sec5on, but it's a bit cumbersome to have it organized this way.

We have added a beMer descrip5on of the data analysis in the methods, and included details of the task. (Lines 135-149) 9. Please add the subheading in the result sec5on for clarity.

We have added the subheadings throughout the results.

10. It is advised to indicate the individual data points in graphs to appreciate data distribu5on.

We have added individual data points to most graphs throughout.
