# Supplementary figures and images for: The Orbitofrontal Cortex Is Required for Learned Modulation of Innate Olfactory Behavior
Source: eNeuro. 2024 Oct 18;11(10):ENEURO.0343-24.2024. doi: 10.1523/ENEURO.0343-24.2024 (PMC11493560; doi:10.1523/ENEURO.0343-24.2024)

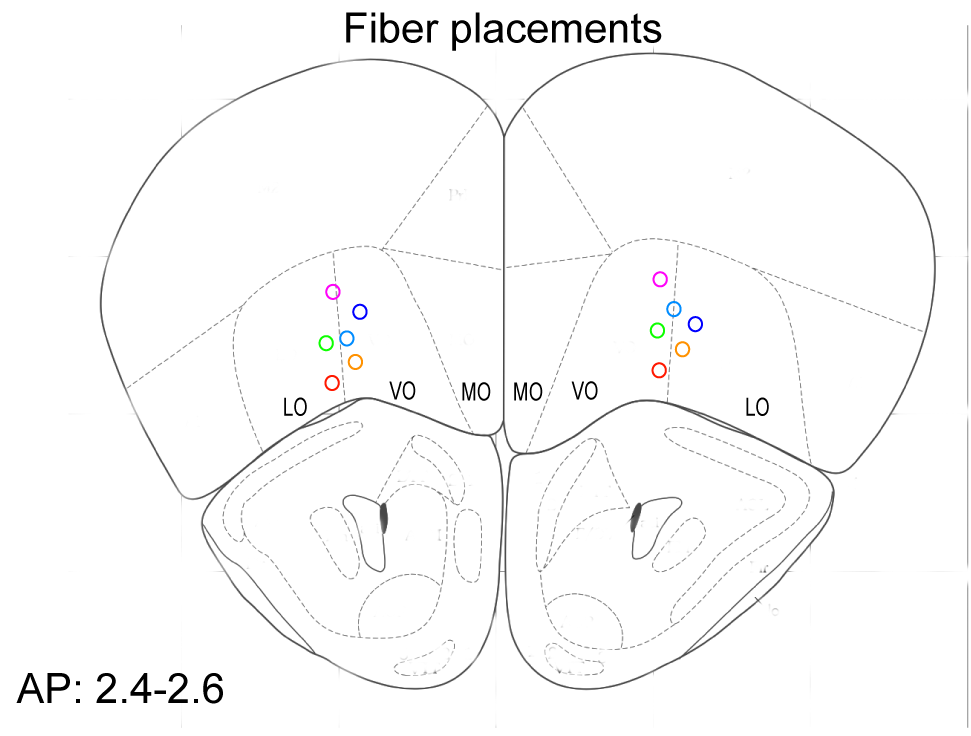

Supplement: Figure 2-1 — Targeting for optogenetic experiments. Schematic of the OFC at approximately 2.5 mm anterior to bregma according to Franklin and Paxinos, which is the midpoint along the anterior-posterior axis. Colored circles indicate the placement of bilateral fibers in each of six animals, whereby one color represents each animal. Fibers were annotated at approximately 2.4-2.6 mm from Bregma. The subregions of OFC are outlined as medial (MO), ventral (VO) and lateral (LO). The fibers were targeted to the boundry of VO and LO and it is estimated that the optical illumination spread 0.4 mm radius from the fiber tip. Download Figure 2-1, TIF file. [file eneuro-11-ENEURO.0343-24.2024-s002.tif]

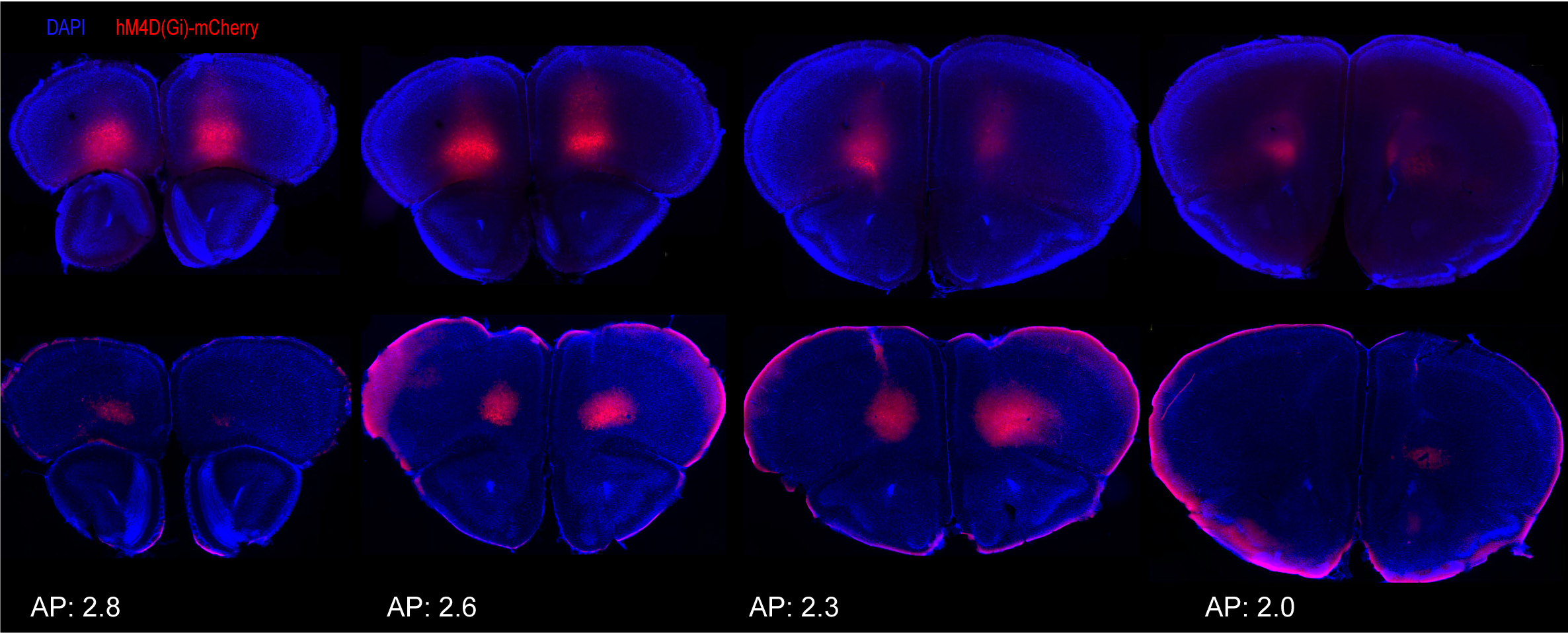

Supplement: Figure 3-1 — Targeting for chemogenetic experiments. Representative histology from bilateral expression of hM4D(Gi)-mCherry in the OFC Mice along the most of the extent of OFC from the anterior (2.8 mm anterior to bregma) to the posterior (2.0 mm anterior to bregma). The top and bottom represent the extremes for viral spread, whereby the top set of images are from on mouse with the largest spread of virus, and the bottom are from a mouse with the smallest spread. Note, the red signal around the perimeter of some bottom images is background fluorescence, or imaging artifact, not mCherry expression. Download Figure 3-1, TIF file. [file eneuro-11-ENEURO.0343-24.2024-s003.tif]
